# Modeling phenotypic heterogeneity towards evolutionarily inspired osteosarcoma therapy

**DOI:** 10.1038/s41598-023-47412-1

**Published:** 2023-11-17

**Authors:** Darcy L. Welch, Brooke L. Fridley, Ling Cen, Jamie K. Teer, Sean J. Yoder, Fredrik Pettersson, Liping Xu, Chia-Ho Cheng, Yonghong Zhang, Mark G. Alexandrow, Shengyan Xiang, Mark Robertson-Tessi, Joel S. Brown, Jonathan Metts, Andrew S. Brohl, Damon R. Reed

**Affiliations:** 1 Adolescent and Young Adult Program, H. Lee Moffitt Cancer Center and Research Institute, Tampa, FL USA; 2 Department of Individualized Cancer Management, H. Lee Moffitt Cancer Center and Research Institute, 12902 Magnolia Drive, Tampa, FL 33612 USA; 3 Biostatistics and Bioinformatics, H. Lee Moffitt Cancer Center and Research Institute, Tampa, FL USA; 4 Molecular Genomics Core Facility, H. Lee Moffitt Cancer Center and Research Institute, Tampa, FL USA; 5 Molecular Oncology, H. Lee Moffitt Cancer Center and Research Institute, Tampa, FL USA; 6 Integrative Mathematical Oncology, H. Lee Moffitt Cancer Center and Research Institute, Tampa, FL USA; 7 Cancer Biology and Evolution, H. Lee Moffitt Cancer Center and Research Institute, Tampa, FL USA; 8 Sarcoma Department, H. Lee Moffitt Cancer Center and Research Institute, Tampa, FL USA; 9 Molecular Medicine Program, H. Lee Moffitt Cancer Center and Research Institute, Tampa, FL USA

**Keywords:** Tumour heterogeneity, Bone cancer, Cancer genomics, Cancer microenvironment

## Abstract

Osteosarcoma is the most common bone sarcoma in children and young adults. While universally delivered, chemotherapy only benefits roughly half of patients with localized disease. Increasingly, intratumoral heterogeneity is recognized as a source of therapeutic resistance. In this study, we develop and evaluate an in vitro model of osteosarcoma heterogeneity based on phenotype and genotype. Cancer cell populations vary in their environment-specific growth rates and in their sensitivity to chemotherapy. We present the genotypic and phenotypic characterization of an osteosarcoma cell line panel with a focus on co-cultures of the most phenotypically divergent cell lines, 143B and SAOS2. Modest environmental (pH, glutamine) or chemical perturbations dramatically shift the success and composition of cell lines. We demonstrate that in nutrient rich culture conditions 143B outcompetes SAOS2. But, under nutrient deprivation or conventional chemotherapy, SAOS2 growth can be favored in spheroids. Importantly, when the simplest heterogeneity state is evaluated, a two-cell line coculture, perturbations that affect the faster growing cell line have only a modest effect on final spheroid size. Thus the only evaluated therapies to eliminate the spheroids were by switching therapies from a first strike to a second strike. This extensively characterized, widely available system, can be modeled and scaled to allow for improved strategies to anticipate resistance in osteosarcoma due to heterogeneity.

## Introduction

With prevalent loss of TP53 and other tumor suppressors, osteosarcoma has a complex genotype profile which varies widely from patient to patient^1–6^. Bulk sequencing characterizations described recurrent targetable copy number alterations, but effective targeting of these gains remains largely unrealized^4^. The impact of immunotherapy in osteosarcoma to date has also been modest^7^. Clinically, osteosarcoma is treated with surgical resection both with neoadjuvant and adjuvant chemotherapy with methotrexate, doxorubicin, and cisplatin (MAP). At present, we lack the tools to change therapy in a patient-specific manner except upon treatment failure and disease progression, though ongoing circulating tumor DNA work may show prognostic and predictive relevance with several ongoing studies. Tumor necrosis is seen in surgical resection specimens after neoadjuvant chemotherapy and correlates with long-term outcome, however alterations in therapy based on necrosis rate have not improved outcomes^8,9^.

Our approach conceptually to osteosarcoma heterogeneity is to consider two populations: (1) a dominant, large population of cells that are mainly in the primary tumor, sensitive to MAP chemotherapy, or amenable to surgery and (2) a minor, resistant population in or outside the resected tumor and insensitive to MAP chemotherapy which leads to relapse. With the ability to often reduce the viable numbers of osteosarcoma cells by two orders of magnitude or more with systemic therapy, and with the often quick and multifocal pulmonary recurrences in the era of amputation alone for therapy, subclinical populations have long been known to be the determinants of outcome in osteosarcoma^10,11^. The vast majority of localized patients and oligometastatic patients achieve a complete response after chemotherapy and surgery. First strikes like surgery and MAP chemotherapy greatly reduce and eliminate the dominant population, but by this definition second strikes are needed to eliminate the minor, resistant population^12^. Historically, a first strike of surgery alone has been inadequate for optimal outcomes, most commonly due to rapid development of pulmonary metastases. Some of these patients could be salvaged with chemotherapy and thoracotomy, strongly suggesting that chemotherapy is able to eradicate microscopic metastases and thus be an effective second strike to surgery alone in about half of osteosarcoma patients^10,11^. A renewed focus on tumor heterogeneity with the dynamics of small, resistant populations is increasingly being realized in the lab^13–15^. Meaningful perturbations of these small populations are more difficult to investigate with typical clinical trial designs^16–18^. Furthermore, single cell sequencing of clinical samples is revealing that resistant, subclinical clones present at diagnosis can be selected for with standard first-line chemotherapy ultimately leading to resistant and fatal disease^13,15,19^.

With the acceptance of MAP chemotherapy into the first strike for all pediatric and Adolescent and Young Adult (AYA) patients, focus has shifted to identifying agents that can improve upon the first strike or become an effective second strike. Unfortunately, early phase trial metrics such as response rate and progression free survival rate depend more on the therapeutic sensitivity of the dominant population. Thus, we lack adequate clinical endpoints and methods to identify all efficacious second strikes. There have been increasing insights into small dynamic populations that resist chemotherapy, undergo epigenetic and phenotypic changes, and can adapt to the lung microenvironment and lead to metastases^13,20^.

Cell lines can be thought of as having significantly reduced heterogeneity compared to clinical disease, both within and across patients^21^. We thus aimed to use co-culture of multiple osteosarcoma cell lines to model osteosarcoma heterogeneity including minor populations. We first characterized genomic features of the cell lines through RNAseq to determine RNA expression and single nucleotide variations (SNVs), and through copy number arrays to assess copy number variation (CNV). As the genomic characterization was highly consistent with prior publications, we then shifted to analysis of the phenotypic features of osteosarcoma cells in different environments, including manipulation of pH and nutrients and exposure to chemotherapeutic agents. Finally, by coculturing the fastest growing and slowest growing cell lines at different ratios, we explored therapeutic schedules and combinations of agents towards complete response where either the fast-growing or slow growing population predominated.

## Results

### Expression and CNV

To characterize genomic variation, we performed a full genomic characterization of the 6 osteosarcoma cell lines. Pediatric sarcomas, particularly osteosarcomas, are known for having considerable rearrangements associated with genomic instability^6,22^. RNA, DNA, and miRNA from each of the osteosarcoma cell lines were isolated for expression, gene fusions, single nucleotide variations (SNVs), and copy number variation (CNV) analysis, using human osteoblasts as a control (Supplemental Fig. 1). Our results were consistent with previous findings of a complex genomic landscape. Overall, osteosarcoma cell lines typically demonstrated a trend toward copy number gains^6,23^. Among a list of selected genes, both confirmed and putatively associated with osteosarcoma, only 9 copy number losses were observed out of 66 (11 genes across 6 cell lines, Supplemental Fig. 1B). Inactivating changes in the osteosarcoma-associated oncogene TP53, a primary driver of genomic instability, were confirmed in 5 of 6 cell lines—homozygous missense (R156P) mutations in 143B and MNNG, intron 1 translocations in MG63, OS252, and SAOS2, while U2OS is TP53 wildtype (Supplemental Fig. 1C).

### Growth kinetics

We next evaluated the growth of the 6 osteosarcoma cell lines and analyzed the resulting growth curves in order to derive basic kinetics such as growth rate and doubling time (Supplemental Fig. 2). 143B was the fastest growing culture with doubling times as low as 9 h with little noticeable impact of contact inhibition occurring prior to 48 h. SAOS2 was the slowest growing culture and appears to be subject to significant levels of contact inhibition due to doubling times slowing considerably during the log-phase period of 24–48 h as cell concentration increases.

### Competition and nutrient deprivation

**Figure 1:**
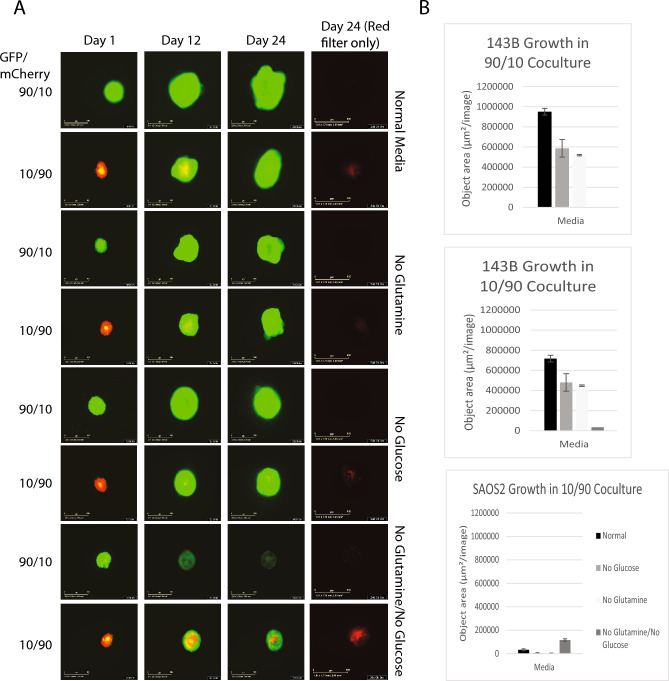
Spheroid growth under nutrient deprivation. (**A**) 143B (green) cells and SAOS2 (red) cells were cocultured in in “normal” media (4.5 g/L glucose, 2.5 mM L-glutamine, pH 7–7.4 and antibiotics), no glutamine media, no glucose media, or no glutamine or glucose media for 24 days. Spheroids were done in triplicate containing 90% of one cell line and 10% of the opposite cell line. First three panels include red and green filters while fourth panel is red filter alone to show persistence of SAOS2 growth. (**B**) Bar graphs showing the growth of 143B in different media types at day 24. No graph exists for SAOS2 in 90/10 coculture because cells were outcompeted and driven to extinction by 143B. Third panel shows persistence of SAOS2 cells in 10/90 coculture.

Under coculture conditions, when 143B was given an advantage in numbers (90% 143B vs. 10% SAOS2, the 90/10 coculture), 143B was able to outgrow and therefore outcompete SAOS2, rendering it undetectable even when deprived of nutrients (Fig. 1). However, in the 10/90 coculture (10% 143B, 90% SAOS2), a remnant population of SAOS2 was still present after 24 days (Fig. 1B, bottom panel). While a low pH (6.5) modestly affected the growth of 143B (Supplemental Fig. 3), high pH had no effect, and pH overall had little effect on the cells. SAOS2 growth under high/low pH was commensurate with growth in normal media. Even when low pH was paired with no glucose, no glutamine, or the absence of both nutrients, growth curves under these conditions showed results similar to nutrient deprivation at normal pH.

Although loss of glucose or glutamine in the media reduced the growth of 143B and drastically reduced growth when both were removed (Fig. 1A), in the 90/10 coculture, 143B is still able to outcompete SAOS2. SAOS2 in single culture was less sensitive to nutrient deprivation and growth decreased slightly only when glucose was removed from the media but was unaffected by the loss of glutamine (Supplemental Fig. 3B). This differential response to glutamine stood out as a selection pressure parameter to explore further. Most notably, however, was that in the 10/90 coculture, a remnant population of SAOS2 was able to persist in all media types (Fig. 1A, right column).

### Chemical screen

Having established growth phenotypes and characterizing genotypes, we were interested in characterizing the selective pressures of potential therapies by a chemical screen. In continuation and consolidation with our previous work assessing efficacy of FDA approved and experimental chemotherapeutic compounds against osteo- and other sarcomas, we assayed an additional 100 agents bringing the total number of drugs for which we have screened against osteosarcoma to 131 (Supplemental Table 1)^24^.

**Figure 2:**
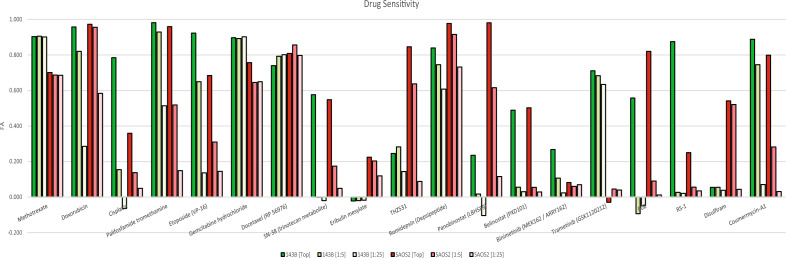
Chemical Screen. Drug sensitivities for 143B and SAOS2 cell lines at top concentration, 1/5 the top concentration, and 1/25 of the top concentration.

Approximately 30 agents in our screen demonstrated sufficient activity (Fraction Affected (FA) > 0.5), while also being differentially active against 143B versus SAOS2 (Supplemental Fig. 4). Several FDA approved compounds demonstrated selective cytotoxicity or growth inhibition of 143B (trametinib, cisplatin, gemcitabine) or SAOS2 (panobinostat, disulfiram) along with experimental small molecules preferentially inhibiting 143B (RS-1) or SAOS2 (THZ531) (Fig. 2, see Supplemental Fig. 5 for results from all 6 cell lines). 143B and SAOS2 responded readily to current clinically utilized drugs such as doxorubicin, cisplatin and 4HC (active metabolite of cyclophosphamide), with some selectivity of methotrexate observed consistent with findings from a number of other studies (Supplemental Fig. 6)^24,25^.

### 3D coculture chemical screen

**Figure 3:**
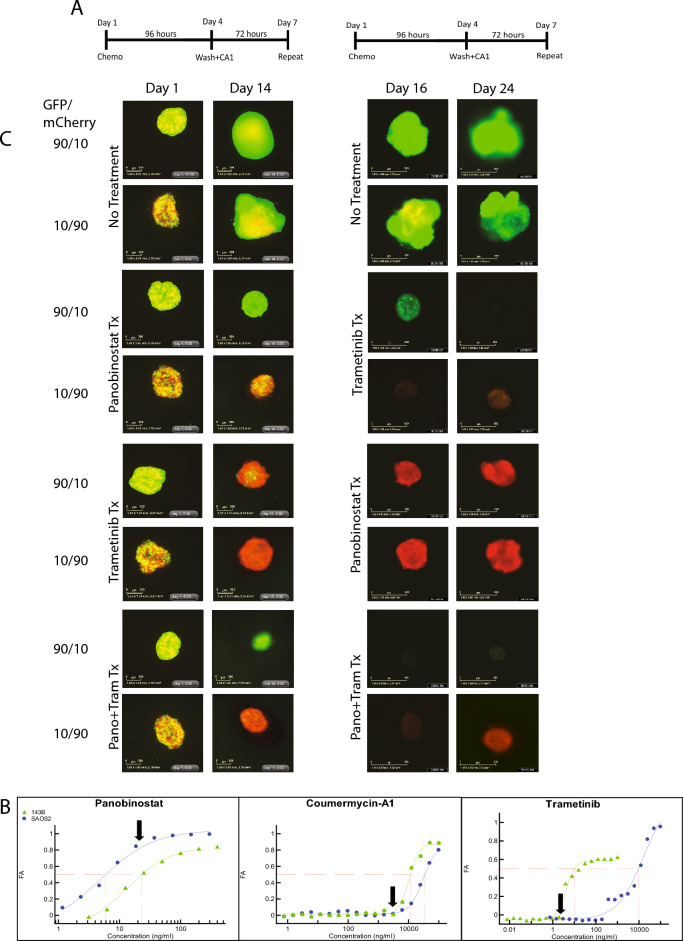
(**A**) Schematic of treatment for spheroids. Spheroids were treated with chemotherapy for 96 h, washed and treated with CA1 for 72 h, and then the process was repeated. On day 16, spheroids were treated with opposite drug (from trametinib to panobinostat and vice versa) for 96 h, then with CA1 for 72 h, and then the process was repeated until the experiment was terminated. (**B**) Single agent dose response plots for panobinostat, trametinib, and CA1. Dose response plots for 143B and SAOS2 plotted as Fraction Affected (FA) versus concentration (ng/ml). Data recovered after 72 h of drug treatment with plotted points representing the mean FA for four technical replicates. Black arrows indicate concentration used in drug treatment of spheroids. (Trametinib: 22.2 ng/ml, Panobinostat: 15 ng/ml, CA1: 5.7ug/ml). Red dashed lines indicate IC50 for each cell line. Note the differential response between cells line for panobinostat and trametinib. (**C**) Coculture Panobinostat and trametinib treatment. Coculture of 143B (green) and SAOS2 (red) cells in spheroids using treatment regimen described above. Treatment with panobinostat and then trametinib successfully eliminates both cell lines at 24 days. Treatment with trametinib and then panobinostat successfully eliminates 143B cells but fails to eliminate SAOS2 cells leaving a robust spheroid. Combination therapy at the start is also able to eliminates all cells when 143B cells predominate to start but considerations for toxicity may affect translation depending on the agents being investigated.

**Figure 4:**
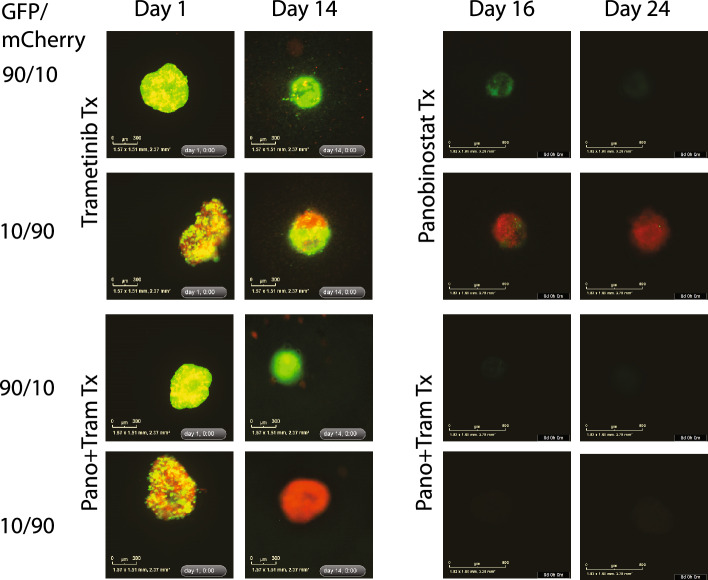
Coculture under nutrient deprivation. 143B (green) and SAOS2 (red) cells in spheroids grown in media containing no glutamine. 143B and SAOS2 cells both continue to persist at day 24 of treatment.

With these considerations in mind, we selected 143B to represent the dominant and first strike sensitive osteosarcoma cell type in our heterogeneity model and the SAOS2 to represent a resistant, minor clone. We selected the drugs identified above that showed a differential sensitivity to these cell lines and configured dosing regimens we anticipated would favor the survival of one cell line over the other. The regimens were constructed sequentially to allow combination or sequential therapy timing to be explored towards clinical trial translation with a 96-h treatment followed by 72-h of recovery (Fig. 3A). We observed that cells were eventually able to recover from treatment and as a result, we included coumermycin-A1 (CA1) a replication CMG-helicase inhibitor^26^ during the recovery time to affect the cell cycle and limit S-phase (Supplemental Fig. 7). Treatment was done at clinically achievable levels for all drugs used (Fig. 3B). Treating concurrently with panobinostat and trametinib was the most effective regimen for eliminating both cell types (Fig. 3C). Treating with panobinostat first eliminated SAOS2 cells leaving a small remnant of 143B cells that are then eliminated by trametinib treatment despite their being less sensitive to it. In addition, though 143B was not as sensitive to panobinostat treatment, once the treatment was switched to trametinib, 143B was eliminated in all cocultures. Conversely, treating with trametinib initially successfully eliminates 143B from coculture. However, SAOS2 is now unaffected by treatment with panobinostat, leaving a robust spheroid after multiple treatment regimens. We hypothesized that by adding glutamine deprivation to the chemotherapy regimen utilized above we would be able to eliminate 143B faster providing a more robust treatment and a shorter treatment regimen allowing successful elimination of both cell lines sequentially. Unexpectedly, eliminating glutamine reduced the sensitivity of 143B cells to treatment (Fig. 4). In the 90/10 coculture, 143B cells persisted, and both SAOS2 and 143B cells survived in the 10/90 coculture. We thus could only eliminate the coculture cells by sequencing therapy affecting 143B and SAOS2 cells simultaneously or by eliminating SOAS2 as the minor clone with panobinostat then using trametinib on the resultant 143B population.

## Discussion

We modelled osteosarcoma heterogeneity leveraging a phenotypic characterization of growth and sensitivity to a large panel of agents allowing us to translate therapies that affect both lines, or to preferentially select for 143B or SAOS2 cells. This ability to drive the model towards enrichment for one or the other population allowed for a better understanding of sequencing and scheduling of multiple therapies demonstrating both the need for multiple agents and suggesting the earlier introduction of the second agent after a first strike vastly reduces the cancer cell population and is more efficacious than delaying a second strike^12,27,28^. This warrants further exploration and would support the hypothesis that maintenance strategies should be moved closer to the start of therapy than what is currently being explored in sarcoma trials.

Bulk sequencing has not yet identified a clear therapeutic target in osteosarcoma. Our model is composed of cell lines with extensive RNAseq and copy number characterization which will be deposited in public databases to explore correlations between agents and molecular changes. We found more value in model development from characterizing the phenotype of growth in optimized media. Adjusting nutrients, pH, and media changes allows for modelling of more dynamic systems and environments with this same concept^29^. With faster growth in optimized conditions, we considered the 143B cell line to be akin to the dominant population initially present at diagnosis and often more sensitive to chemotherapy which aligns with reported in vivo data^30^. Another way to conceptualize these cell lines is that 143B is a “cream skimmer” that is more fit in times of optimal environmental conditions while SAOS2 is a “crumb picker” able to adapt to suboptimal conditions, including chemotherapeutic selection, and thus increases fitness relative to 143B at suboptimal times. This “cream skimmer” and “crumb picker” concept has many ecological examples for competing populations^31,32^. Panobinostat in this model thus becomes an agent required to eliminate a subclinical clone of SAOS2 when present at 10% in spheroid. It is important to note that elimination of such a minor population would not be detected by using radiographic response rates in conventional clinical trials. A better understanding of therapy effects on subpopulations would improve our chances to identify therapeutic strategies to improve survival for patients with osteosarcoma. Serial assessments of resection specimens, paired relapse specimens and ctDNA will hopefully improve on our ability to address subclinical populations of osteosarcoma which often determine the ultimate outcome^33–35^.

Solid tumors like osteosarcoma are more complex and dynamic than we appreciate clinically with fixed chemotherapy plans. Heterogeneity, along with our inability to measure and therapeutically leverage competing clones can explain why we haven’t made sufficient progress in the cure rates for osteosarcoma in four decades. Since we can more easily measure the dominant clones in osteosarcoma with existing techniques such as bulk sequencing, response rate, and event free survival, we focus on areas of disease best addressed by surgery at the expense of understanding small, residual populations below limits of radiologic detection^1–6,36^. We do not currently knowingly take advantage of intratumoral competition, do not design studies to prevent emergence of resistant clones, nor have a good understanding of how individual agents affect subpopulations of tumors^13,20,37,38^. Single cell sequencing and focused studies on early metastases are improving our understanding of osteosarcoma biology and providing insights regarding why we have failed to improve on its outcome^13,15,20,37^.

Despite the enthusiasm for sequencing, we believe incorporating tumor phenotype into treatment planning holds at least the same promise for translating towards successful trials in osteosarcoma. We recognize the importance of heterogeneity at presentation as reflected in our model and do support continued evaluations of genomic sequencing at diagnosis and resection to better characterize clinical heterogeneity. We thus present a proposed synthesis of the genomic and phenotypic worldview of shared osteosarcoma changes. Copy number variations, which vary across patients but are remarkably stable over the course of therapy and progression, suggest that much of the evolution after diagnosis is epigenetic^37^. But most of these DNA changes are likely noise, and osteosarcoma can be conceptualized by altering a handful of important and well characterized yet undruggable cancer pathways. TP53 loss (evolvability contributing to increased adaptations)^6^, RAS pathway gains (foraging and intake of carbohydrates and amino acids)^2^, an abnormal cell cycle with CMG helicase wild type status (DNA replication and never genes)^39–41^, and MYC (resistance)^13,42^ capture common and recurrent genetic changes in osteosarcoma. Until we find agents to meaningfully alter these pathways, multiple strategies will be needed to tackle the resulting complex phenotype. We believe robust and high throughout preclinical systems will be necessary to prioritize such multistep strategies. We propose that this model of heterogeneity can help experimentally evaluate concepts related to a therapeutic strategy based on evolutionary tumor dynamics.

## Methods

### Cell lines

In this study we evaluated 6 established osteosarcoma cell lines (U2OS, MG-63, OS252, SAOS2, 143B, MNNG). U2OS, MG-63, SAOS2, 143B, and MNNG were obtained from ATCC. OS252 was a gift from Richard Gorlick at MD Anderson^43^. The identities of the lines were confirmed using STR analysis. Cells were maintained in DMEM + 15% FBS and antibiotics. Basic growth characteristics and kinetics including doubling time and log-phase were determined at several starting cell concentrations.

### Cell viability assays

Screening methodology, described in prior publications^24,44^, was utilized to explore monoculture and co-culture growth characteristics under environmental stress or in the presence of chemotherapeutic agents. Cell viability was quantified using Cell-Titer Glo 2.0 or Real Time Glo (CT-Glo, RT-Glo, Promega, Madison, WI, USA). Following addition of CT-Glo to cell cultures, cells were agitated for 30 min on a shaker at room temperature. Luminescence was measured using a Cytation 3 plate reader (Bio-Tek Instruments) at room temperature or at 37 °C for CT-Glo or RT-Glo, respectively. Raw data were transferred to Microsoft Excel workbooks, where subsequent background subtraction and normalization analyses were conducted.

### High throughput viability assays (2D)

Cells (2.7 × 10^3^) were seeded onto 384-well plates, with subsequent additions of drug treatments (when applicable) and CT-Glo, using a Precision XS liquid handling station (Bio-Tek Instruments, Winooski, VT, USA). Cells were grown 24 h prior to the addition of any chemical agents and were subjected to drug or vehicle control for 72 h before being assayed with CT-Glo. All drug screens were completed in 2D using cell monolayers. For high throughput characterization of cell growth across multiple cell lines concurrently, several plates for each cell line were seeded with a series of cell concentrations ranging from 1,800 to 47,000 cells/cm^2^ (based on surface area, 1:1.5 serial dilution). Viability was quantified every 24 h until growth curves plateaued at around 96 or 120 h. Each experimental or control condition was split into a minimum of 4 technical replicates. Biological replicates were averaged following normalization.

### RNA-seq, expression analysis, and SNV analysis

Cell lines at 80% confluence were subjected to RNA-sequencing for gene expression analysis. Total RNA was prepared from each osteosarcoma cell line using the *mir*Vana miRNA Isolation Kit (Cat# AM1560, Invitrogen, Thermo Fisher) and RNA-sequencing libraries were prepared using the Nugen Ovation Human FFPE RNA-seq Multiplex System according to the manufacturer’s protocol (Tecan Genomics, Inc, Redwood City, CA). Library size and quality were assessed using the Agilent BioAnalyzer (Agilent Technologies, Santa Clara, CA, USA) and sequencing was performed using the NextSeq 500 v2 sequencer (150 cycles) to generate a targeted 80 million 75-base paired-end reads. Raw sequence data were demultiplexed using the Illumina bcl2fastq2 software (Illumina, Inc., San Diego, CA, USA). Paired-end RNAseq reads were preprocessed for quality assessments and adapter trimming and aligned to the human reference genome hs37d5 using TopHat v2.0.13 default setting^45^. Gene-level expression was quantified using HTSeq v0.6.1^46^ based on the RefSeq gene model downloaded from USCS Table Browser. Normalized read counts were obtained via calculating library size factors and scaling raw read counts using the Bioconductor R package DESeq2 v.1.6.3^47^. microRNA-Sequencing was performed using the Illumina TruSeq Small RNA Sample Preparation Kit, which enables the sequencing of small RNAs between 17 and 35 nucleotides. Briefly, universal RNA adapters were ligated to 1 ug of total RNA, which was used to generate single-strand cDNA following the manufacturer’s protocol (Illumina, Inc., San Diego, CA). Following PCR amplification and gel purification, the final libraries were reviewed on an Agilent BioAnalyzer DNA 1000 chip and were then sequenced on the Illumina NextSeq 500 sequencer to generate approximately 10 million sequencing reads per sample.

To detect driver mutations in the RNAseq data, sequence reads were aligned to the reference human genome (hs37d5) with STAR and duplicate identification, insertion/deletion realignment, split and trim, quality score recalibration, and variant identification were performed with the Picard toolkit (http://broadinstitute.github.io/picard/) and Genome Analysis ToolKit (GATK, Broad Institute, Cambridge, MA, USA)^48,49^. Sequence variants were annotated to determine genic context (i.e., non-synonymous, missense, splicing) using ANNOVAR^50^. Additional contextual information was incorporated, including allele frequency in other studies such as 1000 Genomes, the NHLBI Exome Sequence Project, in silico functional impact predictions, and observed impacts from databases like ClinVar (http://www.ncbi.nlm.nih.gov/clinvar/) and the Collection of Somatic Mutations in Cancer (COSMIC).

### Copy number variation (CNV) and analysis

Copy number variation (CNV) and loss-of-heterozygosity (LOH) status were obtained with the CytoScan HD Assay (Affymetrix, Santa Clara, CA, USA), which was performed on each cell line starting with 250 ng of DNA. DNA was extracted using the Blood and Cell Culture DNA Midi Kit (Qiagen, Hilden, Germany). The CytoScan HD assay uses 750,000 SNP probes and 1.9 million non-polymorphic probes to report genome-wide copy number aberrations at a resolution of 25–50 Kb. In addition, the assay can measure genome-wide LOH, including copy-neutral LOH. The data generated from the assay were normalized, copy number status calculated, and the data reviewed for quality using the Chromosome Analysis Suite (ChAS) v3.0 (Thermo Fisher Scientific, Waltham, MA).

### Nutrient depletion

Twelve different conditions were assessed at notated concentrations (10, 50, 90, 100% (or single culture)) with an initial spheroid size of 1000 cells/well (with 3.5% Matrigel, Corning, Inc., Corning, MA). Media containing 4.5 g/L glucose, 2.5 mM L-glutamine, pH 7–7.4 and antibiotics was deemed “normal” media. Subsequent conditions included no glucose (0 g/L), no glutamine, and no glucose or glutamine. Due to prior work and the importance of tumor microenvironmental conditions the lab was interested in varying pH on the cells and the effects of the small molecules tested, As a results we included conditions with high pH (8.0), low pH (6.5), high pH with no glucose, low pH with no glucose, high pH with no glutamine, low pH with no glutamine, high pH with no glucose or glutamine, and finally low pH with no glucose or glutamine media.^31^ Media was changed every ~ 3 days. Plates were incubated in an Incucyte SX5 where brightfield and fluorescent scans were taken every 24 h.

### Chemical screen

Drug screening with cells in log phase was conducted using phamocokinetic guided dose levels when available and high-throughput cell viability assays as previously described^24^. Briefly, we utilize a system to evaluate combinations with the aim of rapidly translating that data into clinical trials^24^. We selected FDA-approved agents or agents with strong preliminary data for OS, clinically achievable dosages, and tolerable dosing schedules. Treated experimental wells were normalized to untreated wells for a given cell line. In the case of these earlier agents without consistent human or animal drug data, we chose 3 fixed concentrations for each agent. Drugs were then selected based on differential sensitivity to either the 143B or the SAOS2 cell line for further use.

### Coculture (3D spheroids)

The 143B and SAOS2 cell lines were transduced with GFP lentivirus (Cat#: PLV-10002-200, Cellomics Technology, Halethorpe, MD, USA) and mCherry lentivirus (Cat#: LVS(VB220407-1474cpn), VectorBuilder, Chicago, IL, USA) respectively, according to manufacturer’s protocol. The GFP/mCherry containing cells were selected with 5 µg/ml of puromycin. Cells were then plated on Greiner 96-well U-bottom, Cellstar® cell-repellent plates (Greiner Bio-One, Kremsmünster, Austria) using 3.5% Matrigel (Corning, Inc., Corning, MA) to form spheroids. Spheroids were characterized with an initial spheroid size of 1,000 cells/well comprised of SAOS2 alone, 143B alone and SOAS2 comprising 10, 50, or 90%, with 143B cells comprising the remainder to 100%.

### Drug-treated spheroids

Spheroids again at notated SAOS2 compositions (10, 50, 90, 100% (or single culture)) with a total cell number of 20,000 cells/well to start with a larger spheroid were treated for 96 h with panobinostat or trametinib or both in normal media or media with no glutamine at clinically achievable levels based on available pharmacokinetics from clinical trials. Cells were then treated for 72 h with Courmermycin-A1 (CA1), a Cdc45-MCM-GINS helicase inhibitor (CMGi) that helps prevent cell recovery after treatment, and then the whole cycle was repeated. Plates were incubated in an Incucyte ZOOM system where brightfield and fluorescent scans were taken every 12 h.

### Supplementary Information

Supplementary Information 1.

Supplementary Information 2.

## Data Availability

The datasets generated and analyzed during the current study are available in the GEO repository at the following links: RNAseq: https://www.ncbi.nlm.nih.gov/geo/query/acc.cgi?acc=GSE240278 (reviewers can use token kvidcakcjfqprohv to access the data). CNV: https://www.ncbi.nlm.nih.gov/geo/query/acc.cgi?acc=GSE240546 (reviewers can use token slkdkeaazvubnwn to access the data).
