# Supplementary material for: Modeling phenotypic heterogeneity towards evolutionarily inspired osteosarcoma therapy
Source: Sci Rep. 2023 Nov 17;13:20125. doi: 10.1038/s41598-023-47412-1 (PMC10656496; doi:10.1038/s41598-023-47412-1)
Supplement: Supplementary file 1 — Supplementary Information 1. [file 41598_2023_47412_MOESM1_ESM.pdf]

Welch, et. al., Supplemental Figure 1: Genomics

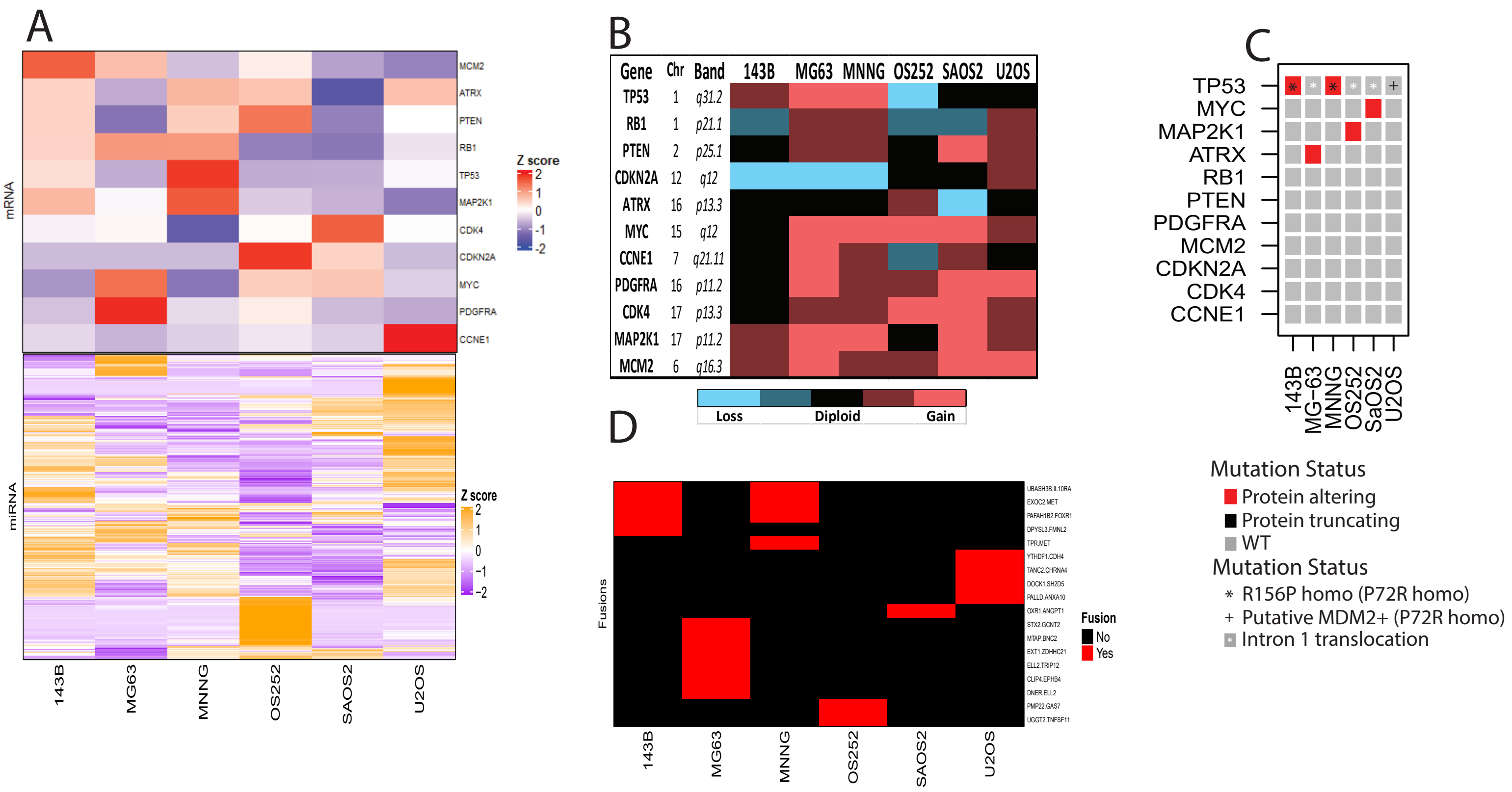

Supplemental Figure 1: Genomics of osteosarcoma cell lines for genes of interest. (A) Heatmap for osteosarcoma cell lines revealed by RNA-seq analysis of mRNA and miRNA. (B) CNVs in 6 osteosarcoma cell lines delineated by chromosome demonstrating diploid, gain, or loss. (C) Oncoprint for protein-altering mutations in 6 osteosarcoma cell lines. Grey boxes indicate "wild type" (at least one reference call was made at a mutated position), red boxes indicate a protein altering mutation, and black boxes indicate protein truncation. In addition to mutation calls for TP53, mutation type is also designated by a black plus sign (putative MDM2+, P72R homo status), a black star (R156P homo, P72R homo status) or a white star (intron 1 translocation). (D) Selected gene fusions detected.

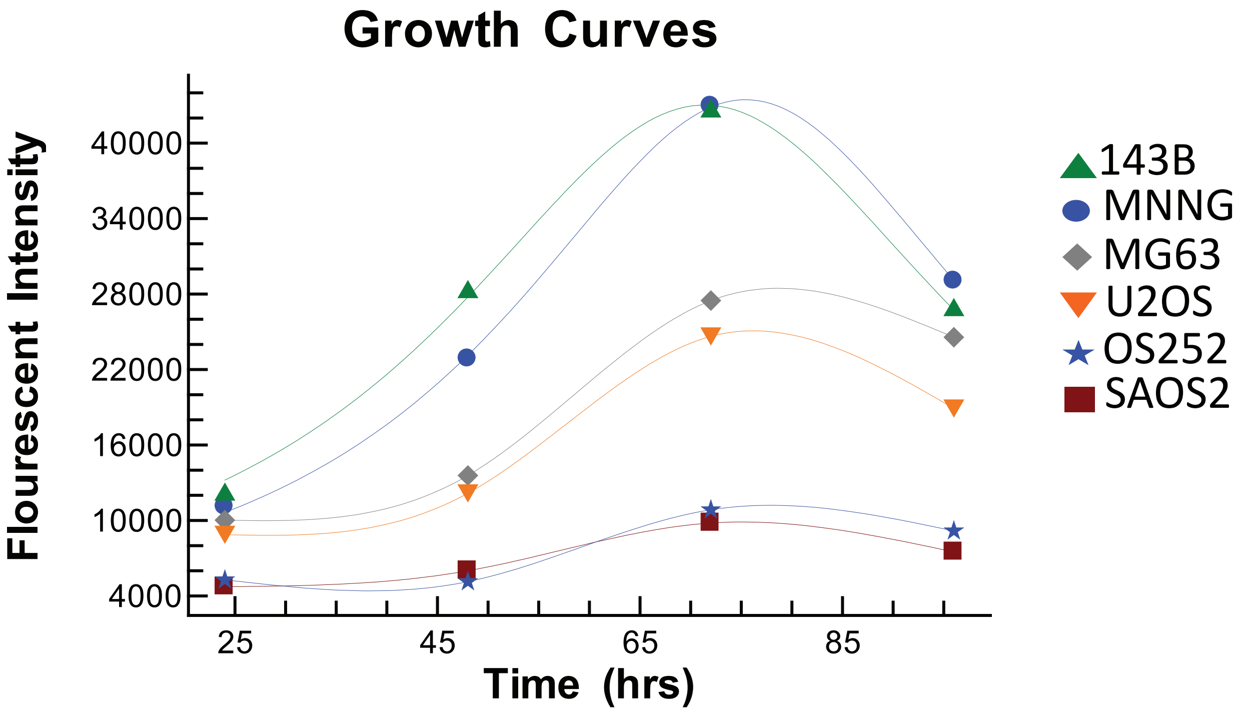

Supplemental Figure 2: Growth kinetics of osteosarcoma cells in mono-culture. Osteosarcoma cell lines were assessed at 2700 starting cell concentration (cells/cm^2). Doubling times between the period of 24 and 48 hours were mathematically derived from growth curve data.

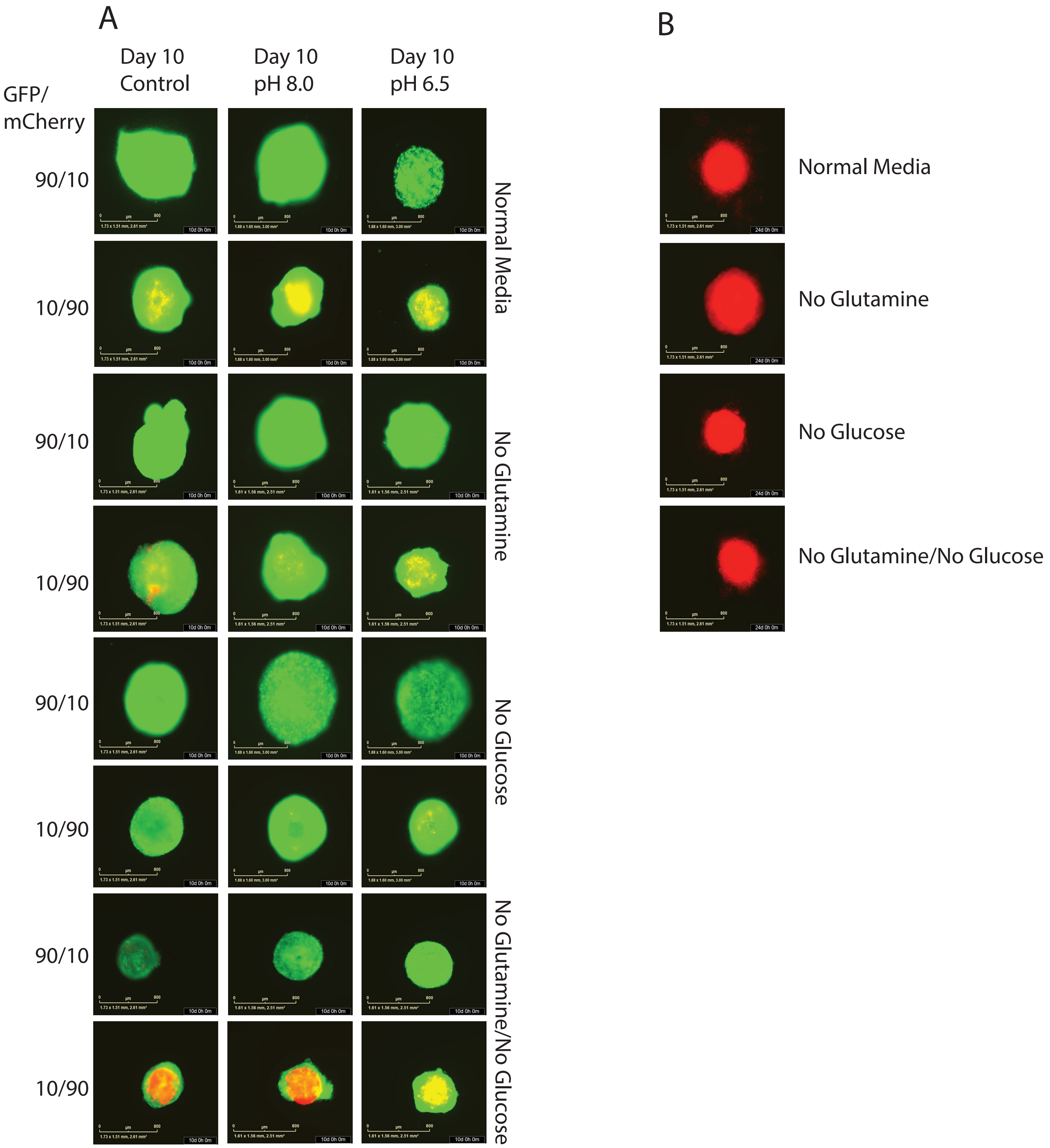

Supplemental Figure 3: (A) Growth kinetics of osteosarcoma cells in high and low pH media. 143B (green) cells and SAOS2 (red) cells were cocultured in high and low pH media with normal nutrients ((4.5g/L glucose, 2.5mM L-glutamine, pH 7-7.4 and antibiotics), no glutamine, no glucose, and no glutamine or glucose media for 10 days. Spheroids were grown in triplicate. (B) Growth of SAOS2 under nutrient deprivation. Spheroids containing only SAOS2 cells were grown in normal media, no glutamine media, no glucose media, and no glucose or glutamine media in triplicate. Being starved

Supplemental Figure 4: Differential Sensitivity

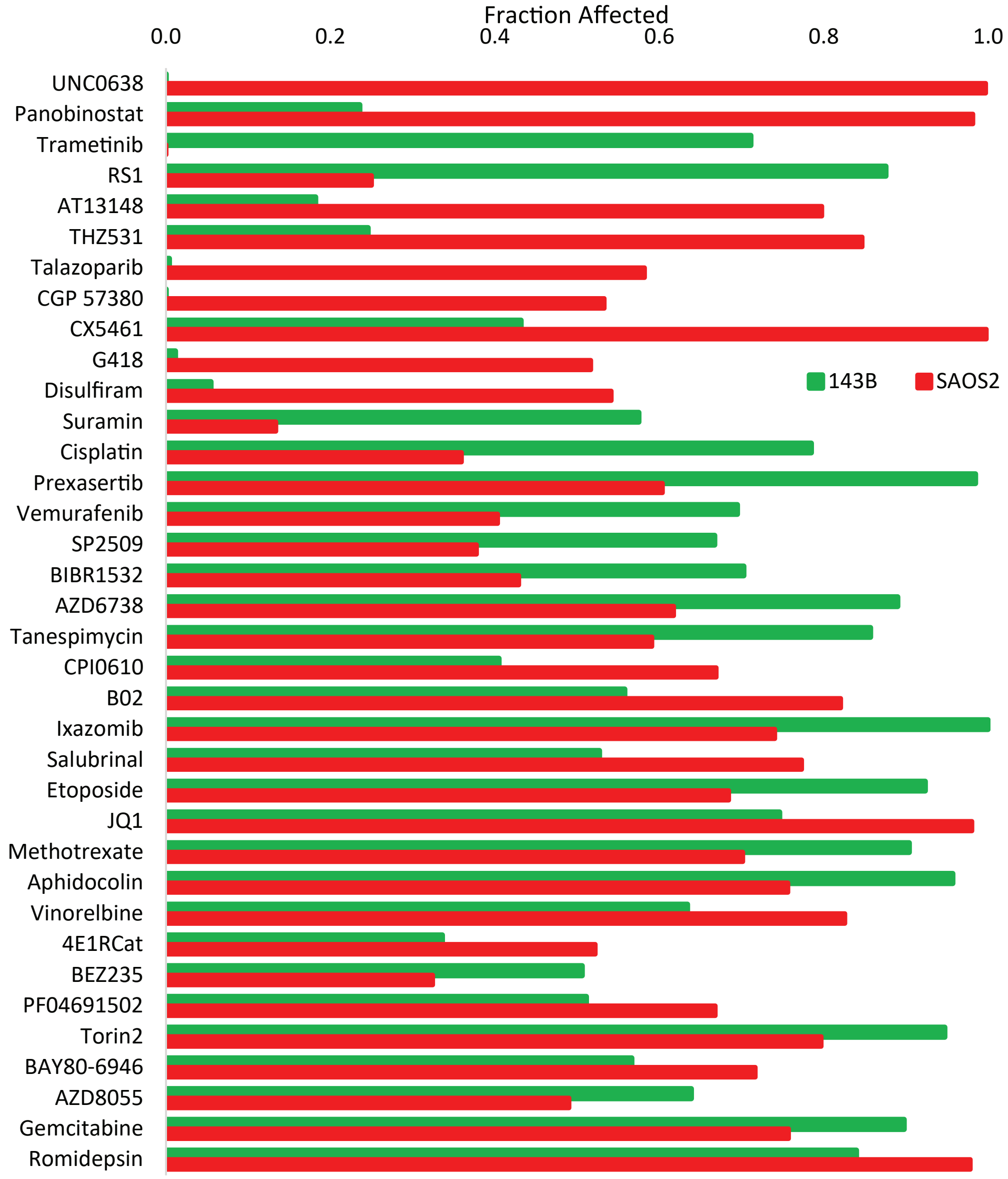

Supplemental Figure 4: Differential sensitivity of chemotherapeutic, targeted, and experimental agents to 143B and SAOS2 cell lines. Graph of agents with an FA>0.5 in either 143B or SAOS2 sorted by standard deviation to demonstrate absolute sensitivity differences between the two cell lines.

Supplemental Figure 5: Drug Screen

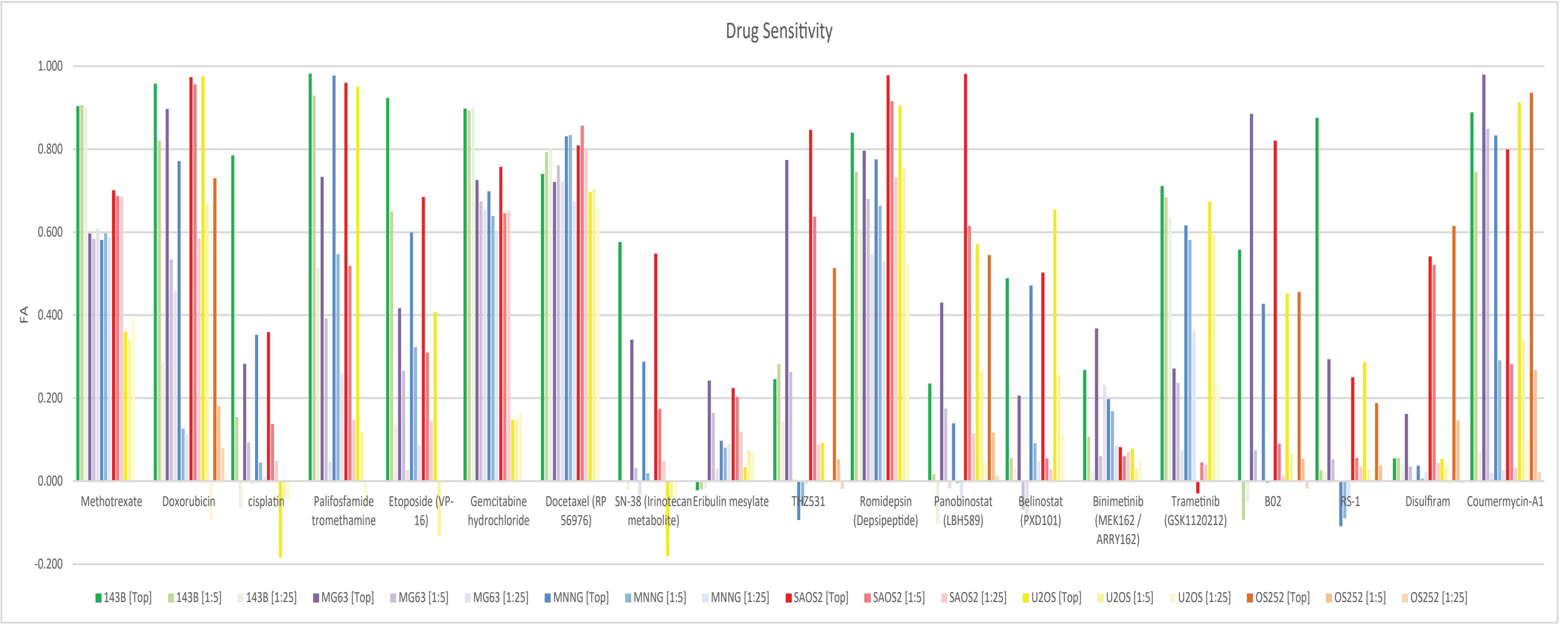

Supplemental Figure 5: Drug Screen. Top agents of interest were tested in 6 osteosarcoma cell lines at 3 concentrations.

Supplemental Figure 6: MAP Dose Response Curves

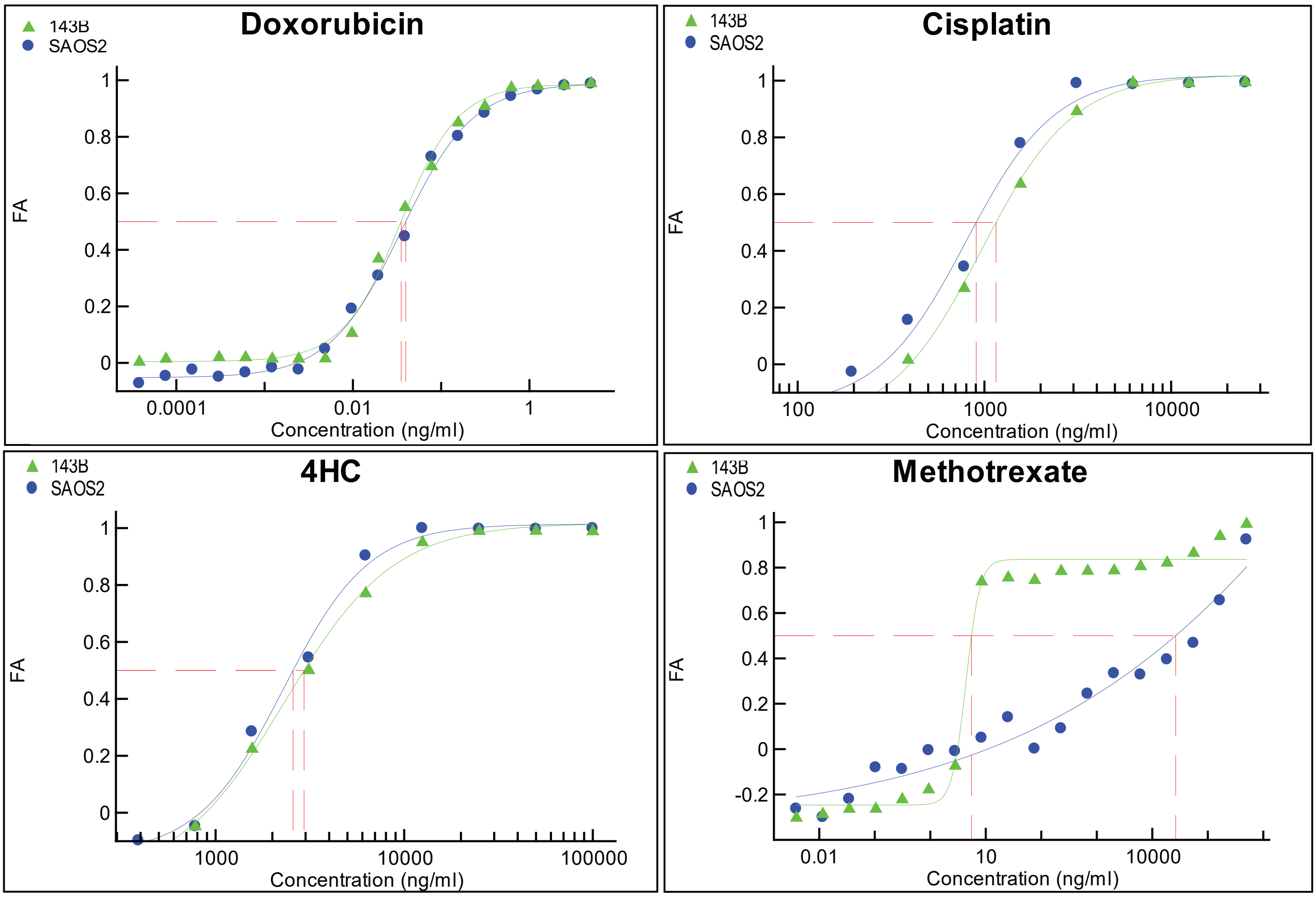

Supplemental Figure 6: Single agent dose response plots for Doxorubicin, methotrexate, cisplatin, and 4-hydroperoxycyclophosphamide (4HC). Dose response plots for 143B and SAOS2 plotted as Fraction Affected (FA) versus concentration (ng/ml). Data recovered after 72hrs of drug treatment with plotted points representing the mean FA for four technical replicates.

Supplemental Figure 7: CA1

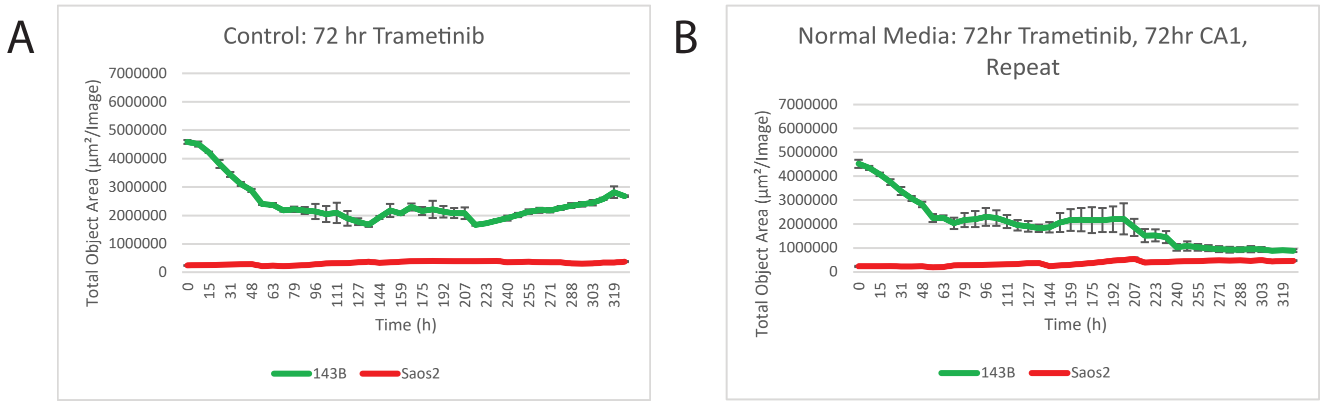

Supplemental Figure 7: (A) 143B cell recovery. Graph of 143B cells recovering from trametinib treatment over time. (B) CA1 prevention of 143B cell recovery. Graph showing the lack of recovery of 143B cells during trametinib treatment with the addition of CA1 treatment. Graph also indicates increased response to treatment.
